# Entropic and Complexity Measures in Atomic and Molecular Systems

**DOI:** 10.3390/e25020367

**Published:** 2023-02-17

**Authors:** Juan Carlos Angulo, Sheila López-Rosa

**Affiliations:** 1 Departamento de Física Atómica, Molecular y Nuclear, Universidad de Granada, 18010 Granada, Spain; 2 Instituto Carlos I de Física Teórica y Computacional, Universidad de Granada, 18010 Granada, Spain; 3 Departamento de Física Aplicada II, Universidad de Sevilla, 41004 Sevilla, Spain

Both entropy and complexity are central concepts for the understanding and development of Information Theory, playing an essential role in the increasingly numerous applications in a huge diversity of fields. Adequate methods of quantifying the entropy of physical systems and processes have been the object of design of numerous researchers for more than a century, usually based on a probabilistic description using discrete or continuous variables depending on the analysis framework considered. In this sense, Shannon entropy deserves a special mention as a pioneering measure; this has also given rise to the subsequent birth of, e.g., the Rényi and Tsallis entropies, or the relative entropy between distributions in close connection with the ‘mutual information’ among descriptive variables.

The quantitative handle of the notion ‘complexity of a system’ (or process) arose much more recently, in the mid-1990s. The pioneering measure of complexity (LMC, in accordance with the names of its authors) includes the Shannon entropy, together with the distance to equiprobability or ‘disequilibrium’. Among the representative properties for any appropriate measure of complexity, it reaches its minimal value in the limits of extremal order and disorder. Complementary to LMC complexity, additional product-like complexity measures have been subsequently defined. Let us emphasize recent generalizations in terms of Rényi entropies, as well as those enclosing the content of gradient of the distribution as a local property to determine the searched complexity.

This Special Issue encloses different applications of information-theoretical concepts for the analysis of physical systems, based on their specific descriptors and variables. The aforementioned applications consider both theoretical and numerical analyses, some of them for arbitrary dimensionality, and the results are systematically interpreted in terms of physically relevant magnitudes and concepts, for instance: energy, correlation, coupling, uncertainty, and reactivity, among others.

Some specific physical systems have been considered in the present Special Issue. Such is the case of many-fermion systems, at times generically handled by means of solvable models [1], allowing a comparison between different systems regarding their respective information-theoretic measures. Particularly for few fermion systems, the above study allows one to interpret their differences from a structural perspective. In this sense, the behavior of the lowest lying energy states provides emergent information on system complexity.

Another kind of multifermionic system is the atomic systems, here considered for a wide range of electron numbers. An appropriate description requires the use of realistic models to handle an appropiate wavefunction, making possible the simultaneous study of the position and momentum conjugated spaces [2]. Taking into account that most atomic systems are structured as a nucleus surrounded by a diversity of electrons, it is pertinent to ask about the influence of any arbitrary electron on each of the others within the set, and consequently about the eventual structural changes in the system when adding or removing an electron. These questions are addressed by considering the interelectronic ‘mutual information’ of the pair distribution and the ‘quantum similarity’ among different distributions. The conclusion from this is that the well-known atomic shell structure plays a central role in answering the above questions, in contrast with the size or weight of the system under consideration.

In opposition to the previous examples, single-electron atoms (i.e., hydrogenic systems) are also considered in this Special Issue, providing an in-depth analysis of especially interesting frameworks [3]. All this study was performed analytically, for systems of arbitrary dimensionality (including the high-dimensionality limit), and in any state as described by the appropriate quantum numbers. Analytical treatment allows the derivation of multidimensional position–momentum distributions, expressed in terms either of radial expectation values or Shannon/Rényi entropies. This work opens the door to similar studies for more sophisticated multidimensional systems.

Combined theoretical and numerical analyses are particularly interesting, as happens with the work dealing with the entropic study of the transformation from the LS-coupled configuration space to the jj-coupled one [4]. Shannon entropy is shown to be an adequate measure of the purity of the wavefunction for atomic states, as described in different configuration spaces, focusing on the Ni-like ions that constitute the corresponding isoelectronic series. The interpretation of results is provided in terms of eigenlevel anticrossing of adjancent levels.

In spite of the advantages of dealing with specific systems and/or processes, studies affording a generic treatment of many-body systems are always welcome, an example being the information-theoretical description based on the local-energy concept, emphasizing its interpretation for the analysis of molecular systems and chemical reactions [5]. Differences between the classical and quantum-mechanical descriptions are manifest, particularly if quantified by means of Shannon’s global entropy and Fisher’s gradient information.

In summary, these papers constitute a diversity of apportations to the information-theoretic abilities and applications in a variety of physical fields, focusing on fermionic systems as considered from different perspectives, including analytical and numerical ones. The relevancy of entropic and complexity measures is revealed, together with their interpretation in terms of physically relevant properties and concepts. Some results regard both conjugate spaces, different energetic states, and/or arbitrary dimensionality.

## Data Availability

The editorial does not report any data.
